# Impressions and perceived barriers of implementing water-based foam and nitrogen foam swine depopulation methods at the farm level

**DOI:** 10.1371/journal.pone.0354430

**Published:** 2026-08-20

**Authors:** Janice Y. Park, Marie R. Culhane, Magnus R. Campler, Andréia G. Arruda, Andrew S. Bowman

**Affiliations:** 1 Department of Veterinary Preventive Medicine, The College of Veterinary Medicine, The Ohio State University, Columbus, Ohio, United States of America; 2 Department of Veterinary and Biological Sciences, College of Veterinary Medicine, University of Minnesota, St. Paul, Minnesota, United States of America; North-Caucasus Federal University - Pyatigorsk Campus: Severo-Kavkazskij federal’nyj universitet Patigorskij institut filial, RUSSIAN FEDERATION

## Abstract

Animal depopulation is a critical component of emergency planning across U.S. livestock industries. Class A water-based foam (WBF) and high-expansion nitrogen foam (N_2_F) have been researched to help provide the swine industry with alternative tools to perform depopulation at larger scales. At this time, there is little data on perceived advantages or barriers identified by individuals, such as operation owners and employees, who would be closely involved in the initial phase of depopulation. Our aim was to survey farm personnel to gather logistics-focused insight upon viewing WBF and N_2_F demonstrations. Survey questions reflected method-specific difficulties scored using a 4-point Likert scale, along with respondent demographics and free response comments. Select survey sections were analyzed using multivariable linear regression models. Other sections, demographic data, and free response comments are presented descriptively. Data from 51 WBF and 76 N_2_F surveys were available, with 121 responses disclosing career position. Average (± SD) respondent age was 49.9 (± 13.2) years, with 22.3 (± 16.0) years for average experience in the swine industry. Respondents consisted of 42.2% (51/121) veterinarians, 32.2% (39/121) other farm staff, 13.2% (16/121) owners of their organization, 9.1% (11/121) animal handlers, and 3.3% (4/121) classified as “other”. Depopulation method was the only variable of interest associated with differences in scores for performing tasks and method implementation, with N_2_F perceived as more difficult than WBF (*P* < 0.05). Largest proportions of comments for both methods pertained to ease of use and cost. At least one respondent suggested pre-assembled kits or services for either method. The input from a wide range of respondents emphasizes the perceived importance of method practicality for emergency planning. Our findings suggest that method demonstrations alone are insufficient; future work must focus on efforts to meet the need for developing avenues for user education for both WBF and N_2_F.

## Introduction

Depopulation events are distinct from euthanasia in that they address mass emergencies, which can involve hundreds or thousands of animals, making this process logistically challenging. Since depopulation efforts in the United States (U.S.) will inevitably involve producers and their employees, the methods used must be adaptable, accessible, and user-friendly. Despite the increasing potential for foreign and emerging animal diseases and other unforeseen catastrophes, the U.S. swine industry has few available options that satisfy these criteria. Carbon dioxide gas (CO_2_) has been utilized at larger scales in swine [1] and is listed as “preferred” under the 2019 Guidelines for the Depopulation of Animals published by the American Veterinary Medical Association (AVMA) [2]. Recently, this has been classified under “Tier 1” (highest priority) under the AVMA’s most recent updates to these Guidelines in 2026 [3]. However, the availability of additional options remains vital to circumvent situations where the CO_2_ gas supply is limited during widespread emergencies [4,5] or if producers lack access to CO_2_-specific equipment.

Medium-expansion Class A water-based foam (WBF) and high-expansion nitrogen foam (N_2_F) are relatively novel technologies that would facilitate swine depopulation at larger scales. Water-based foam consists of a 1% solution of Class A foam concentrate in water, which is expanded to a ratio of 40–50:1 foam to water and applied over the tops of pigs held in a containment system. Once the pigs are completely covered with foam, WBF mechanically obstructs the proximal airway structures in pigs [6], rapidly reducing gas exchange and leading to unconsciousness and death. On the other hand, N_2_F is infused with nitrogen gas (N_2_), which results in the generation of a high expansion (>300:1) foam comprised of bubbles containing >98% N_2_ gas. For the HEFT AB system, N_2_F is delivered into the animal holding container via screened openings, and pigs are covered in foam as it rises to the top of the container. The bubbles are broken down using an applied pulse of N_2_ gas [7]. One alternate system for N_2_F delivery into an animal holding container is to apply N_2_F over the tops of pigs using hoses and nozzles, fully covering them with N_2_F. This relies on animal movement to break down the bubbles (AES Inc. system) [8]. For both N_2_F systems, anoxic conditions are created as N_2_ is released from the foam bubbles, displacing the surrounding oxygen (O_2_) to levels below 2% [9,10]. Under the new 2026 Guidelines, WBF is listed in the U.S. as Tier 1 for swine [3] upon demonstrating the ability to cause rapid death in pigs of various sizes and age groups [11–13]. Other studies [7,10] found that HEFT AB N_2_F is suitable for depopulating swine and poultry using animal holding containers. Providing multiple options for swine depopulation will provide farms with some flexibility in emergency planning tailored to individual needs, regional resource availability, and logistical preferences.

While research has repeatedly determined the efficacy of WBF and N_2_F, their use may be limited unless individuals involved in emergency response are fully capable of acquiring and correctly deploying these methods. To gauge perspectives of swine industry stakeholders (i.e., pork producers, state and federal regulatory agents, animal scientists, swine veterinarians), upon viewing the process of WBF, Cheng et al. administered surveys pre- and post-WBF demonstrations. They found positive changes in stakeholder perceptions upon viewing WBF in comparison to those expressed prior to the demonstrations [14]. Another study [15] surveyed stakeholders immediately after demonstrating WBF, AES Inc.-based N_2_F, and CO_2_, where respondents evaluated methods based on the AVMA’s criteria for depopulation methods [2]. They determined that respondents expressed preferences for foam methods over CO_2_ across multiple of these AVMA criteria [15].

While prior findings reflect promising end user support for these methods, we have yet to explore how WBF and N_2_F would be perceived in the eyes of farm employees and animal caretakers. Because these personnel are closely familiar with their farm’s day-to-day resources and labor, they could provide novel insight regarding logistical obstacles at the farm level that may otherwise be overlooked. Therefore, the objective of this study was to provide live demonstrations of WBF and the HEFT AB N_2_F system and survey these individuals to gather their perceptions regarding supply accessibility, method execution, and farm-wide integration. This information remains key for detecting areas to address prior to implementing WBF and N_2_F, along with identifying any modifications needed to increase user education and method familiarity.

## Materials and Methods

### Study approval and participant recruitment

This study was determined exempt by The Ohio State University Institutional Review Board (IRB) (Protocol 2024E0666). Participants were recruited during four separate swine depopulation demonstrations. Prior to enrollment, all participants were informed of our maintenance of their anonymity and confidentiality. Informed verbal consent was obtained on an individual basis, and survey(s) were distributed only if an individual provided consent. Any individual who denied consent was not provided survey(s). At least two study team members were present at these events to witness these statements. The performed sequence to obtain consent began with: “…Would you be willing to share your opinions by participating in the survey process? If the respondent said yes: “Thank you so much. We have printed copies of the survey, which you may return to this box once you have completed it. This process is anonymous, so the choice is yours.” If they responded no: “Not a problem. Thank you very much for taking the time to speak with me.” This sequence to obtain verbal consent was approved under the previously noted University IRB.

At the first three events, participants who provided verbal consent were given two printed surveys (one per method). The fourth event was N_2_F-only and had only one survey. Participants observed the generation and deployment of WBF and HEFT AB N_2_F methods on live pigs in containers, as approved through The Ohio State University Institutional Animal Care and Use Committee (IACUC) (Protocol 2020A00000036-R1) and University of Minnesota IACUC (Protocol 2403-41911A). These methods were performed according to protocols previously described in Arruda et al. [12] and Park et al. [6], respectively. Afterwards, survey(s) were completed, and the responses were digitized and stored internally within an institutional two-factor authenticated resource to ensure data protection and confidentiality. Recruited individuals were not compensated for participation.

### Survey design

Two surveys were constructed, consisting of one per method (WBF, N_2_F). The surveys were designed in collaboration with co-authors who have extensive background experience in operating these methods. Question content was based on knowledge of method-specific set ups, along with topics that were identified as potential areas of difficulty for users. Additionally, the content of the demographic questions was formatted to be similar to previous survey-based studies on swine depopulation methods [14,15]. Questions under Sections 1–5 focused on scoring perceived difficulty using a 4-point Likert-scale, with higher scores correlating with greater perceived ease of the task indicated (1 = very difficult, 2 = difficult, 3 = easy, 4 = very easy) (Table 1). Additional questions collected respondent demographic data. The end of the survey provided a blank section for respondents to comment on any foreseen challenges, suggestions for improvement, or provide other feedback. Full details and content of the administered WBF and N_2_F surveys are included under S1 Table.

**Table 1:** Prompts by section for both water-based foam (WBF) and nitrogen foam (N_2_F) surveys. Sections prompting “how difficult” included questions where respondents assigned scores between 1 through 4 (1 = very difficult, 2 = difficult, 3 = easy, 4 = very easy).

Section	Prompt
1	How difficult would it be for your organization to quickly obtain the following mechanical equipment:
2	How difficult would it be for your organization to quickly obtain the following supplies:
3	How difficult would it be for the workers in your organization to perform the following activities:
4	If an emergency occurred today, how difficult would it be for you to *personally perform* the following activities to depopulate swine using the [WBF or N_2_F] method:
5	If an emergency occurred today, how difficult would it be for *your organization* to:
6	What is your position within the organization?
7	What is your current age?
8	How many years have you been associated with the swine industry?

### Data processing and statistical analyses

All statistical analyses were performed using Stata v.19.0 (StataCorp, College Station, TX). Statistical significance was declared as *P* < 0.05. Descriptive statistics were performed on individual questions under Sections 1 and 2, along with demographic variables (position, age, number of years in swine industry). For each response, scores under each section in Sections 3–5, respectively, were averaged. For these sections, the resulting average values were analyzed using separate multivariable linear regression models. Fixed effects of interest included demonstrated method, position within organization, respondent’s age, and number of years in the swine industry. Because N_2_F demonstrations were viewed as either standalone or in conjunction with WBF, the effect of viewing N_2_F demonstrations in series with WBF was investigated using a multivariable linear regression model. For this, whether the N_2_F demonstration was viewed in series was designated as an additional fixed effect of interest. Similar or recurring comments are presented verbatim from respondents.

Because eight survey responses had more than one option selected for Section 6 (*position within organization*), multivariable linear regression models were constructed to see whether the indication of holding multiple positions would have a significant effect on scores. If so, these responses would be recategorized accordingly.

## Results

A total of 52 WBF and 77 N_2_F survey responses were collected across all demonstration events. One participant’s responses for both methods were omitted due to inconsistencies in assigned scores with the corresponding comments, resulting in data available from 51 WBF and 76 N_2_F surveys. Constructed multivariable linear regression models indicated holding multiple positions had no effect on scores for Sections 3–5 (*P* > 0.05). Thus, for the final models, we recategorized these eight responses for Section 6 into the first position the respondents indicated in their respective surveys. Additionally, four other responses had positions outside of the provided selections written in, which were recategorized as “other”, and a total of 6 responses (3 WBF and 3 N_2_F) did not indicate a current position.

Of these surveys, mean (± SD) respondent age was 49.9 (± 13.2) years. Average (± SD) number of years of experience in the swine industry was 22.3 (± 16.0) years. Additionally, 42.2% (51/121) of surveys were completed by veterinarians, 32.2% (39/121) from other farm staff, 13.2% (16/121) from owners of their organization, 9.1% (11/121) from animal handlers, and 3.3% (4/121) from “other” personnel, which were indicated as government or regulatory positions. For all components under Sections 1 and 2, which focused on assessing perceived difficulty of obtaining method-specific components (mechanical equipment, supplies), mean scores for WBF were higher (i.e., perceived as easier) than those of N_2_F (Table 2). Moreover, the most commonly assigned score across all questions under Sections 1 and 2 were “4” for WBF, whereas this was either “3” or “2” for N_2_F.

**Table 2:** Descriptive statistics for Sections 1 and 2 for water-based foam (WBF, N = 51) and nitrogen foam (N_2_F, N = 76) surveys. Values are based on responses scored using a 4-point Likert-scale, with the highest possible value (score = 4) indicating very easy to obtain the component and lowest value (score = 0) meaning very difficult to obtain the component.

Method	Component	Mean	SD	Min	Max	Mode
**WBF**	Water pump	3.70	0.51	2	4	4
	Hoses	3.65	0.56	2	4	4
	Nozzles	3.24	0.80	1	4	4
	Hardware	3.52	0.74	1	4	4
	Foam concentrate	3.42	0.61	2	4	4
	Water and tank	3.57	0.61	2	4	4
	Mixing container	3.51	0.62	2	4	4
	Animal chamber	3.27	0.76	1	4	4
**N**_**2**_ **F**	Gas regulator	2.61	0.92	1	4	2
	Animal chamber	2.11	0.87	1	4	2
	Hardware	2.73	0.88	1	4	3
	Nitrogen tanks	2.47	0.73	1	4	3
	Foam concentrate	2.44	0.75	1	4	2
	Mixing container	2.96	0.86	1	4	3

Method (WBF vs. N_2_F) was the only variable of interest associated with a significant difference in perceived difficulty of performing method-specific activities (Sections 3–4) and difficulty for respondent’s organization to implement the method in question (Section 5) (*P* < 0.001, Table 3). Furthermore, mean scores for N_2_F decreased by 0.41 (*P* = 0.04) and 0.50 (*P* = 0.04) points for Sections 4 and 5, respectively, if observed in conjunction with WBF compared to when viewed standalone, also accounting for position, age, and years in the swine industry.

**Table 3:** Estimated marginal means and descriptive statistics for each method (WBF = water-based foam, N_2_F=nitrogen foam) from multivariable linear regressions for Sections 3-5. Method was the only fixed effect associated with significant differences in average scores in all Sections 3-5. Assigned values ranged from 1 = very difficult to 4 = very easy.

Section	Method	Marginal means	SE	95% CI
3) Organization performing activities	WBF	3.32	0.09	[3.14, 3.49]
	N_2_F	2.86	0.07	[2.71, 3.00]
4) Personally performing activities	WBF	3.27	0.09	[3.10, 3.45]
	N_2_F	2.44	0.07	[2.30, 2.59]
5) Implementing method into organization	WBF	3.26	0.11	[3.05, 3.48]
	N_2_F	2.39	0.09	[2.22, 2.56]

Overall, the largest proportions of returned comments were related to ease of use and expense. Of all WBF surveys, 19.6% (10/51) commented on the practicality of the WBF system (“very simple, low tech”), and 5.9% (3/51) noted the lower “cost of equipment [being] a plus”. However, there were worries about the use of WBF, with 5.9% (3/51) respondents listing the potential for “political limitations” or that their “main concern is...ethical consideration[s]”. For N_2_F, the most identified challenge was the higher cost of the N_2_F specialized system, such as respondents stating, “cost of equipment is the constraint.” This was noted in 18.4% (14/76) of returned surveys. Method complexity and having “a lot of moving parts” were identified in approximately 17.1% (13/76) of N_2_F surveys. Similarly, others (15.8%, 12/76) noted limitations in their access to N_2_F-specialized equipment, such as N_2_ gas and mechanical components. Moreover, 6.6% (5/76) mentioned the need for improved method efficiency to address the capacity to depopulate large herds. Similar to WBF, 5.7% (4/76) of N_2_F respondents expressed concern about “political implications” or animal welfare complaints. However, only N_2_F received comments (2/76) of “seems very humane”. Additionally, a third person stated that the N_2_F was more humane but followed that statement with a question regarding time required for the N_2_F method, “...looks like research says this is the best possible now from actual death of pig, but is the difference worth the speed of water-based foam[?]”. For both WBF and N_2_F, at least one respondent commented on the need to provide a pre-assembled kit or service with contractor support when using either method.

## Discussion

Our goal for this study was to capture impressions of WBF and N_2_F methods from a particular subset of individuals who are most familiar with the day-to-day activities at their respective farms. While we successfully captured a wide range of respondent demographics across four demonstration events, it is particularly interesting that the only variable that had a significant effect on overall scores was the method observed. Considering that respondents had not seen either method prior to these demonstrations, perceived accessibility may be largely influenced by how one gauges the ability to acquire needed equipment and any training needed to use these purchases. Given N_2_F was scored as more difficult when viewed alongside WBF, the responses substantiate the assertion that simplicity and accessibility are key for emergency planning decisions, regardless of user background. This is consistent with a prior study [15], in which the majority of post-demonstration surveys were completed by mainly a different group of professionals from veterinary medicine, academia, pork organizations, or regulatory positions. Those individuals ranked WBF significantly higher (i.e., “better”) than both N_2_F and CO_2_ in terms of supply accessibility [15]. Unlike our present study, however, investigation of demographic variables identified that respondents from regulatory agencies scored WBF lower than those from unspecified organizational backgrounds [15]. Based on this discrepancy, we should consider pursuing more targeted insight from regulatory personnel to better gauge their feedback on these methods, given these individuals’ major role in guiding emergency response.

A large proportion of written comments that focused on method usability and cost was similar to those from respondents in a similar study, who had commented that WBF appeared easy to deploy, while noting that the N_2_F method seemed to involve “limited resources, equipment… [and] seems to be an expensive option” [15], The findings from both prior and these present surveys suggest that practicality and accessibility may very well be the final determinant of what method will be integrated into each farm’s emergency response depopulation protocols.

Noting the expressed concerns regarding expenses and supply bottlenecks, organizations pioneering foam-based methods for swine depopulation should dedicate increased efforts towards exploring method flexibility and affordability, in addition to the usual considerations of scalability and animal welfare. N_2_ gas research [16–18] and N_2_F studies [10,19] have been conducted to explore more humane methods for euthanasia, as CO_2_ has been found to result in pain and respiratory distress in pigs and poultry [20]. However, the large impetus to develop N_2_ for animal depopulation was the SARS-CoV-2 pandemic, when CO_2_ was in short supply [21,22]. Likewise, the WBF method has undergone additional refinement to improve method efficiency and reduce animal distress behaviors [11]. Thus, alternative methods were used during emergency situations [23], and implementation of WBF or N_2_F could address and overcome supply chain bottlenecks or resource shortages.

Furthermore, WBF studies for swine depopulation were initiated in response to the SARS-CoV-2 crisis [13,24], and since 2020, WBF studies have refined the options to use different varieties of Class A foam concentrate brands, concentrations, and different fill rates [11,25]. Because WBF optimization may have been mentioned to participants during the demonstrations, this could partially explain the relatively higher perceived ease of acquiring supplies and generating WBF. Lastly, we find that method feasibility should be of major consideration for the AVMA during the reviewal periods for swine depopulation methods, given that two of the Guidelines for the Depopulation of Animals criteria already pertain to method reliability and ability to maintain functioning equipment [2].

Interestingly, for both WBF and N_2_F, at least one respondent commented on the need for pre-assembled kits or services to streamline supply acquisition. This raises a novel point that has not been mentioned in previous survey-based studies [14,15]. Multiple components for WBF are already available through the National Veterinary Stockpile [26], along with other firefighting equipment distributors. Nitrogen foam services are currently available through an independent contractor in the U.S. [27] and HEFT AB based in Sweden. While N_2_F systems for on farm euthanasia or scaled up for depopulation may be available in the U.K. and E.U. [28,29], having limited N_2_F service contractors in the U.S. puts American farms at risk of substantial delays in time to depopulation and rapid depletion of availability. It is therefore imperative to develop more direct means for N_2_F to increase accessibility. Because multiple respondents also expressed concerns regarding the N_2_F system’s complexity, organizing efforts to arrange equipment bundles with instructional modules may help bridge the knowledge gap between end users and proper N_2_F deployment.

While the need to ensure animal welfare was less often noted as a concern for the respondents in the study herein, animal welfare remains a key point raised for both methods, but particularly for WBF. While the exact nature of respondents’ welfare concerns was not indicated in their responses, for WBF we infer this may be related to an assumed likeness of WBF to animal drowning. Recently, the mechanism of WBF in swine was elucidated using postmortem examination and CT imaging, which concluded that the mechanism was mechanical occlusion of the proximal airways in swine [6], just as it was determined for poultry species [30]. Moreover, gross lesions in the WBF and N_2_F-terminated pigs were noticeably distinct from those terminated using water submersion [6]. However, the misconception of WBF being identical to animal drowning may still be circulating and should be clarified and data regarding the mechanism of death may help provide clarification.

Additional information that should be integrated into discussion points and education during future demonstrations is data regarding both pig and human behavior during end-of-life events. Behavioral data describing swine welfare during WBF and N_2_F for euthanasia and depopulation has been previously investigated [10,11,31]. In addition, the general pool of recruited participants in pig euthanasia and depopulation studies is likely familiar with CO_2_, a Tier 1 method listed in the AVMA Guidelines [2]. Previous studies have found that respondents expressed preference for both foam-based methods over CO_2_. For example, WBF was scored more favorably post-demonstration as pigs’ times to cessation of movement, and vocalizations were shorter [14]. In a study with several respondents observing CO_2_, the participants commented negatively on the excessive vocalization and longer perceived time of distress [15]. In contrast to CO_2_, pig vocalizations with WBF and N_2_F are muted or minimal, potentially due to some degree of upper airway occlusion from foam, and/or by rapid unconsciousness due to anoxia for N_2_F [6]. With both WBF and N_2_F, the use of foam not only obscures what participants hear but also what they see. The pigs remain covered with foam completely for WBF, or until the N_2_F bubbles are burst for N_2_F. The inability to visualize the pigs at all times may have reduced the welfare concerns for participants, potentially reducing the psychological strain from terminating animals, which has been previously described to occur in animal caretakers and veterinarians [32].

## Conclusions

Overall, the results from this study suggest that WBF and N_2_F are perceived as effective methods for depopulation. However, our target farm-level audience continues to perceive barriers and gaps in understanding that must be addressed to ensure successful method integration and deployment on farm. Survey responses yielded valuable points to consider for future directions, raising the idea that providing field demonstrations alone is insufficient. Developing educational opportunities to facilitate discussion will be critical to building confidence in these methods for emergency planning. For example, workshops focused on supply sourcing and costs, or creating online resources discussing equipment setup, could be valuable ways to guide end users towards readily familiarizing themselves with these methods. It will be equally important to integrate evidence-based findings obtained from prior studies to mitigate ongoing concerns regarding swine welfare, as expressed in survey responses. Through these potential avenues, our goal is to guide an informed decision-making process for swine producers formulating their emergency response plans.

## Supporting information

S1 Table Copy of the survey’s provided for the demonstration of water-based foam and nitrogen-based foam during a simulated foreign animal disease outbreak. (PDF)
