# Supplementary material for: Impressions and perceived barriers of implementing water-based foam and nitrogen foam swine depopulation methods at the farm level
Source: PLoS One. 2026 Aug 20;21(8):e0354430. doi: 10.1371/journal.pone.0354430 (PMC13492756; doi:10.1371/journal.pone.0354430)
Supplement: Supplementary file 1 [file pone.0354430.s001.pdf]

S1 Table. Copy of the survey's provided for the demonstration of water-based foam and nitrogen-based foam during a simulated foreign animal disease outbreak.

Water-based Foam Implementation Survey

Situation: Emergency depopulation of the swine on your farm may become necessary in the event of a foreign animal disease outbreak. Please assess your organization's ability to implement a water-based foam system for depopulation.

Use the following scale to answer questions 1-5.

1	2	3	4
**Very difficult**	**Difficult**	**Easy**	**Very easy**

1. How difficult would it be for your organization to quickly obtain the following mechanical equipment:

**Circle one:**	1	2	3	4	Water pump
	1	2	3	4	Hoses (rigid, fire hose)
	1	2	3	4	Foam nozzle
	1	2	3	4	Hardware to connect hoses, water pump, and foam nozzle

2. How difficult would it be for your organization to quickly obtain the following supplies:

**Circle one:**	1	2	3	4	Water-based foam concentrate
	1	2	3	4	Adequate water supply and holding tank
	1	2	3	4	Container for mixing foam solution
	1	2	3	4	Container for depopulation chamber (modified trailer, enclosed coral, etc)

3. How difficult would it be for the workers in your organization to perform the following activities:

**Circle one:**	1	2	3	4	Assemble all components of the foam delivery system
	1	2	3	4	Mix the foam concentrate with water to obtain the proper dilution
	1	2	3	4	Move or load swine into a designated area or container for depopulation
	1	2	3	4	Properly operate the system to make foam
	1	2	3	4	Adhere to safety protocols when working with swine and around water-based foam

4. If an emergency occurred today, how difficult would it be for you to *personally perform* the following activities to depopulate swine using the water-based foam method:

Circle one: 1 2 3 4 Supervise or direct the depopulation event

1 2 3 4 Procure equipment and supplies

1 2 3 4 Assemble the equipment

1 2 3 4 Operate the foam delivery system

5. If an emergency occurred today, how difficult would it be for *your organization* to:

Circle one: 1 2 3 4 Willingly shift to using water-based foam for depopulation of swine

1 2 3 4 To successfully implement the water-based foam method for depopulation

6. What is your position within your organization?

- o Owner
- o Animal handler
- o Other farm staff or employee
- o Veterinarian

7. What is your current age? __________

8. How many years have you been associated with the swine industry? __________

Comments (e.g. What challenges/hurdles do foresee? What are the gaps in knowledge? How can the system be more streamlined? What else would you need to make this work? What are your suggestions for improvement?)

____

____

____

____

____

____

Nitrogen-based Foam Implementation Survey

Situation: Emergency depopulation of the swine on your farm may become necessary in the event of a foreign animal disease outbreak. Please assess your organization's ability to implement a nitrogen-based foam system for depopulation.

Use the following scale to answer questions 1-5.

**1**	**2**	**3**	**4**
**Very difficult**	**Difficult**	**Easy**	**Very easy**

1. How difficult would it be for your organization to quickly obtain the following mechanical equipment:

Circle one: 1 2 3 4 Compressed gas regulator

1 2 3 4 Customized depopulation chamber
(i.e. airtight, internal foam delivery, bubble burst mechanism)

1 2 3 4 Hoses and hardware to connect gas tank to depopulation chamber

2. How difficult would it be for your organization to quickly obtain the following supplies:

Circle one: 1 2 3 4 Compressed nitrogen gas tanks

1 2 3 4 Nitrogen-compatible foam concentrate

1 2 3 4 Container for mixing foam solution

3. How difficult would it be for the workers in your organization to perform the following activities:

Circle one: 1 2 3 4 Assemble all components of the foam delivery system

1 2 3 4 Mix the foam concentrate with water to obtain the proper dilution

1 2 3 4 Move or load swine into a designated area or container for depopulation

1 2 3 4 Properly operate the system to make foam

1 2 3 4 Adhere to safety protocols when working with swine and around compressed gas

4. If an emergency occurred today, how difficult would it be for you to *personally perform* the following activities to depopulate swine using the nitrogen-based foam method:

**Circle one**:	1 2 3 4	Supervise or direct the depopulation event
	1 2 3 4	Procure equipment and supplies
	1 2 3 4	Assemble the equipment
	1 2 3 4	Operate the foam delivery system

5. If an emergency occurred today, how difficult would it be for *your organization* to:

**Circle one:**	1 2 3 4	Willingly shift to using nitrogen-based foam for depopulation of swine
	1 2 3 4	To successfully implement the nitrogen-based foam method for depopulation

6. What is your position within your organization?

- o Owner
- o Animal handler
- o Other farm staff or employee
- o Veterinarian

7. What is your current age? __________

8. How many years have you been associated with the swine industry? __________

Comments (e.g. What challenges/hurdles do foresee? What are the gaps in knowledge? How can the system be more streamlined? What else would you need to make this work? What are your suggestions for improvement?)

____

____

____

____

____

____
